# Muscle LIM protein/CSRP3: a mechanosensor with a role in autophagy

**DOI:** 10.1038/cddiscovery.2015.14

**Published:** 2015-08-03

**Authors:** M M Rashid, A Runci, L Polletta, I Carnevale, E Morgante, E Foglio, T Arcangeli, L Sansone, M A Russo, M Tafani

**Affiliations:** 1 Department of Experimental Medicine, University of Rome, Sapienza, Rome, Italy; 2 Department of Cellular and Molecular Pathology, IRCCS San Raffaele, Rome, Italy; 3 Consorzio MEBIC, San Raffaele University, Rome, Italy

## Abstract

Muscle LIM protein (MLP) is a microtubule-associated protein expressed in cardiac and muscle tissues that belongs to the cysteine-rich protein (CSRP/CRP) family. MLP has a central role during muscle development and for architectural maintenance of muscle cells. However, muscle cells rely on autophagy during differentiation and for structural maintenance. To study the role of MLP in autophagy, we have used C2C12 mouse myoblasts silenced or overexpressing MLP. Our results show that MLP contributes to the correct autophagosome formation and flux by interacting with LC3 as demonstrated by co-immunoprecipitation and PLA assay. In fact, MLP silencing results in decreased LC3-II staining and absent degradation of long-lived proteins. Moreover, MLP silencing impaired myoblasts differentiation as measured by decreased expression of MyoD1, MyoG1 and myosin heavy chain. Ultrastructural analysis revealed the presence of large empty autophagosomes in myoblasts and multimembranous structures in myotubes from MLP-silenced clones. Impaired autophagy in MLP-silenced cells resulted in increased susceptibility to apoptotic cell death. In fact, treatment of MLP-silenced C2C12 myoblasts and myotubes with staurosporine resulted in increased caspase-3 and PARP cleavage as well as increased percentage of cell death. In conclusion, we propose that MLP regulates autophagy during muscle cell differentiation or maintenance through a mechanism involving MLP/LC3-II interaction and correct autophagosome formation.

## Introduction

Muscle LIM protein (MLP/CSRP3/CRP3) belongs to a family of cysteine-rich protein, which is preferentially expressed in cardiac and skeletal muscle^1,2^ and primarily helps in the development of muscle during early stages of life. Homozygous deletion of MLP in mice has been implicated in the distinct forms of cardiomyopathy and heart failure.^3,4^ MLP has been suggested as a cytoskeletal protein that acts in concert with titin and telethonin as a stress sensor.^5^ MLP is important for myofibril organization and is critical in the process of muscle differentiation and maintenance of the contractile apparatus.^3,6^ It is thought that LIM domain of MLP acts as protein-protein-binding interface.^7^ For example, MLP interacts with several proteins in the Z-disk of sarcomere such as T-cap/Telethonin, zyxin and α-actinin.^8^ Despite the fact that MLP is a cytoskeleton-associated protein that may act as a mechanical stress sensor^9^ activating downstream signaling cascades, a role for MLP in autophagy has not yet been determined. Of note, autophagy is important during differentiation and to maintain muscle mass,^10^ therefore, autophagy regulation by MLP could also explain the important role of this protein for the proper differentiation of muscle cells as well as for the correct turnover of damage organelles or structures in cardiac and skeletal muscle cells.

Autophagy is a bulk degradation process by which cytosolic proteins and damaged organelles are recycled back through the formation of double membranous vesicles, termed autophagosomes and then fusion with lysosomes for the degradation by lysosomal acid hydrolases and proteases.^11^ Under conditions of stress, such as starvation and hypoxia, autophagy is activated, promoting cell survival by releasing energy substrates via degradation of cellular constituents and by eliminating defective or damaged organelles.^12^ Autophagy is an important process to maintain cellular homeostasis during energy deprivation, while it also contributes to the quality control of proteins and organelles during stress. Yeast deficient in autophagy rapidly die under nutrient-poor condition suggesting its important function to preserve nutrient supply.^13^ The process of quality control autophagy is critically important for terminally differentiated, longer lived cells such as neurons and myocytes.^14^ Autophagy also plays important roles in cellular remodeling during differentiation and development of multicellular organisms such as fly, worm and slime mold.^15^ Studies have shown that autophagy has important role in muscle maintenance and to restore muscle integrity by using muscle-specific inactivation of autophagic genes.^16,17^ Autophagy is implicated in muscle homeostasis, removing protein aggregates and abnormal organelles, which would otherwise lead to muscle toxicity and dysfunctional muscle. Deficient autophagy has been suggested to contribute in different forms of inherited muscular dystrophies, including Bethlem myopathy, Ullrich congenital muscular dystrophy and in Duchenne muscular dystrophy (DMD).^18^ Impaired autophagy is also seen during acute and chronic phases of cardiac hypertrophy.^19,20^ Although the functional significance of autophagy in cardiac hypertrophy is not fully understood, autophagy may help in the clearance of damaged proteins and organelles. Autophagy emerges thus as an essential process for the clearance of defunct cellular organelles and a continued inhibition of autophagy exaggerates dystrophic phenotype.^21^ Autophagy inhibition is thought to play a role in many myopathies with inclusions or with abnormal mitochondria.^22,23^

The present study investigates, in muscle cells, the involvement of MLP in the regulation of the autophagic process. In particular, we show that by binding to LC3 (microtubule-associated protein 1 light chain 3), MLP controls autophagy in both C2C12 myoblasts and myotubes thereby playing a major role in muscle differentiation and maintenance. Finally, as also reported in other systems, autophagy impairment due to MLP silencing results in increased apoptosis in both myoblasts and myotubes.

## Results

### MLP role in skeletal muscle differentiation

**Figure 1:**
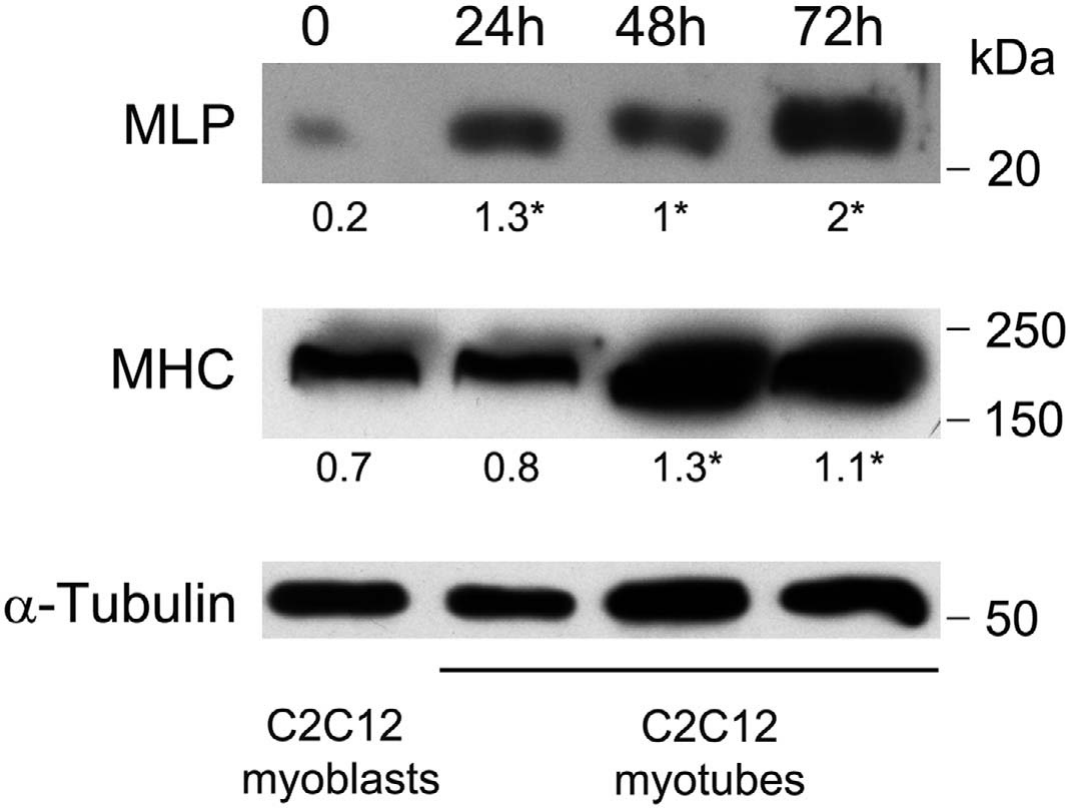
MLP expression during differentiation. C2C12 cells were grown as myoblasts. Alternatively, C2C12 cells were differentiated into myotubes for 24, 48 and 72 h as described under Materials and Methods. MLP and MHC expression was measured as described under Materials and Methods. *α*-Tubulin was used as loading control. Densitometric analysis was performed with ImageJ and average values from three separate experiments are reported below the bands. *Significantly different from myoblasts. Significance was set at *P*<0.05.

MLP is a mechanotransductor with a central role for cardiac and skeletal muscle structure maintenance.^1,3^ To explore the expression of MLP during myogenic differentiation, we used C2C12 cells, a well-established cell line that is derived from mouse satellite cells and faithfully mimics the skeletal muscle differentiation process *in vitro*. Increased MLP expression was observed after 24, 48 and 72 h in C2C12 cells in the presence of differentiation medium (Figure 1). Such MLP increase was concomitant with increased expression of the differentiation marker myosin heavy chain (MHC; Figure 1).

**Figure 2:**
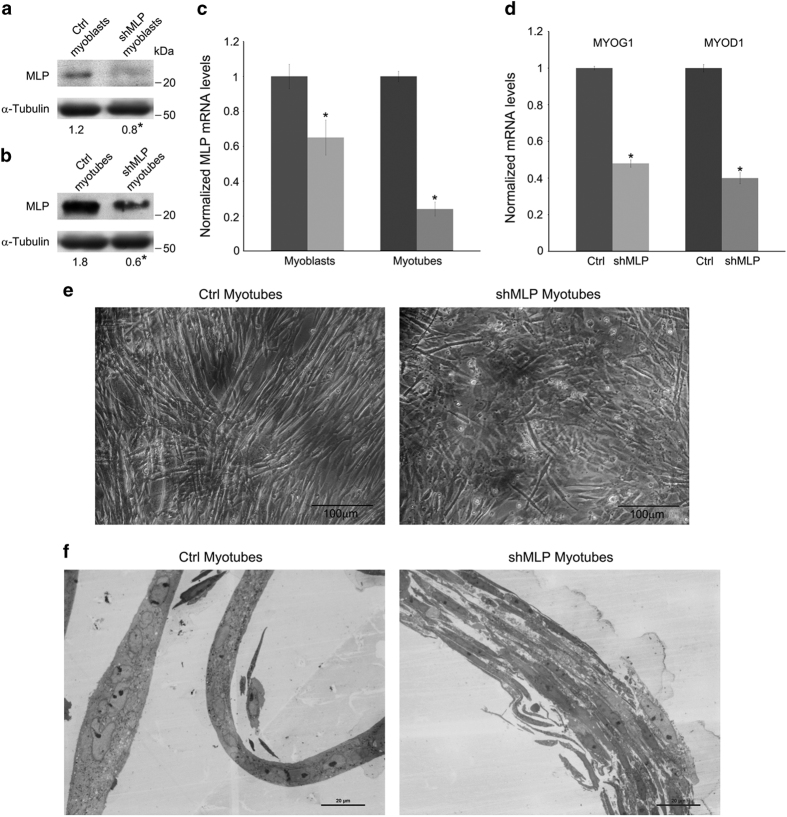
MLP silencing reduces muscle cell differentiation. (**a** and **b**) C2C12 cells were transfected with MLP-specific shRNA and non-targeting scramble plasmid (ctrl) and then differentiated for 72 h. MLP expression was measured in myoblasts and myotubes as described under Materials and Methods. *α*-Tubulin was used as loading control. Densitometric analysis was performed with ImageJ and average values from three separate experiments are reported below the bands. *Significantly different from ctrl myoblasts or myotubes. Significance was set at *P*<0.05. (**c**) Downregulation of MLP mRNA in MLP-silenced myoblasts and myotubes measured by real-time PCR. MLP mRNA from ctrl cells was set at 1. (**d**) MyoG1 and MyoD1 mRNA expression in myotubes from ctrl and shMLP cells measured by real-time PCR. MLP mRNA from ctrl cells was set at 1. (**e**) Images from ctrl and MLP-silenced cells differentiated as described under Materials and Methods. Formation of elongated myotubes is observed in ctrl cells but is missing in MLP-silenced cells in which many rounded cells are present. (**f**) Optical microscopy images of fixed and epon-embedded myotubes prior to electron microscopy analysis. During differentiation (72h), ctrl cells form multinucleated myotubes while MLP silencing cells have defective myotube formation with fiber-like disorganized structure. *Statistically significant from ctrl cells. Significance was set at *P*<0.05.

To investigate MLP function in myogenic differentiation, we used shRNA directed against MLP to deplete its presence in C2C12 myoblasts and then monitored morphological differences during C2C12 differentiation in myotubes. MLP silencing in myoblasts and myotubes is shown in Figures 2a and b. MLP silencing resulted in reduced expression of its mRNA (Figure 2c). Of note, MLP silencing impaired C2C12 differentiation in myotubes as documented by a reduction of mRNA expression for MyoG1 and MyoD1 (Figure 2d) and by a disorganization and reduction of myotubes (Figures 2e and f). These results suggest that silencing of MLP reduces myotube formation and inhibits skeletal myogenic differentiation.

### MLP regulates autophagy in skeletal muscle

**Figure 3:**
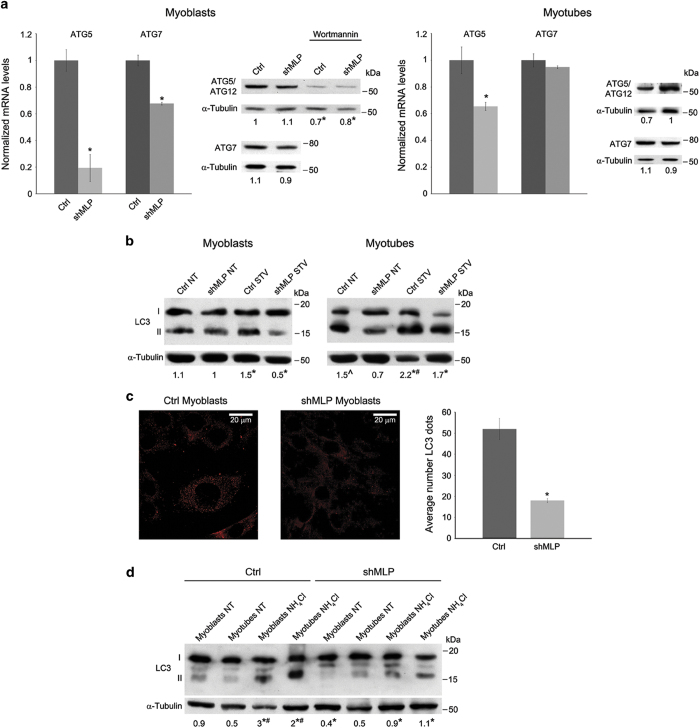
MLP silencing prevents autophagy induction. (**a**) ATG5 and ATG7 mRNA expression in myoblasts (left side) and myotubes (right side) from ctrl and shMLP cells measured by real-time PCR. ATG5 and ATG7 mRNA from ctrl cells was set at 1. Protein expression level of ATG5/ATG12 complex and ATG7 was also measured in myoblasts and myotubes as described under Materials and Methods (left side). Reduction of ATG5/ATG12 expression in the presence of 400 nM wortmannin was determined by western blot as described under Materials and Methods. *α*-Tubulin was used as loading control. Densitometric analysis was performed with ImageJ and average values from three separate experiments are reported below the bands. *Significantly different from control WT or shMLP myoblasts. Significance was set at *P*<0.05. (**b**) Ctrl and shMLP myoblasts and myotubes were either left untreated or kept under starvation for 17 h. LC3-II expression was measured by western blot as described under Materials and Methods. *α*-Tubulin was used as loading control. Densitometric analysis of LC3-II was determined with ImageJ and average values from three separate experiments are reported below the bands. *Significantly different from scramble control (ctrl) or shMLP NT myoblasts or myotubes; ^#^significantly different from starved shMLP myoblasts or myotubes; ^significantly different from shMLP NT myotubes. Significance was set at *P*<0.05. (**c**) Ctrl and shMLP cells were fixed, permeabilized and LC3 expression determined by immunofluorescence as described under Materials and Methods. Average number of LC3 dots in ctrl and shMLP myoblasts were counted and plotted (right side). *Significantly different from scramble ctrl myoblasts. Significance was set at *P*<0.05. Size bars are indicated in the figure. (**d**) Myoblasts and myotubes from ctrl and shMLP cells were either left untreated or treated with ammonium chloride (10 mM) for 2 h. Accumulation of LC3-II in scramble ctrl cells compared to shMLP cells was measured by western blot as described under Materials and Methods. *α*-Tubulin was used as loading control. Densitometric analysis of LC3-II increase was determined with ImageJ and average values from three separate experiments are reported below each band. *Significantly different from NT myoblasts; ^#^significantly different from NH_4_Cl-treated shMLP myoblasts or myotubes. Significance was set at *P*<0.05.

Maintenance of skeletal muscle structure and function requires efficient and precise metabolic regulation.^24^ Autophagy plays a key role in metabolic homeostasis of diverse tissues such as skeletal muscle.^25,26^ To investigate whether MLP silencing in skeletal muscle affects autophagy induction, we measured transcripts of autophagy genes in either myoblasts or myotubes from control and MLP-silenced cells. In myoblasts, we observed a significant mRNA decrease for ATG5 and ATG7 in MLP-silenced cells compared to control (Figure 3a). In myotubes, such a difference was still present for ATG5 but not for ATG7. Moreover, compared to myoblasts, myotubes from MLP-silenced cells showed an increase in mRNA levels for ATG5 and ATG7 (Figure 3a). However, we did not observe any difference in ATG5 and ATG7 protein expression in myoblasts and myotubes from WT or MLP-silenced cells probably due to some post-transcriptional regulatory pathway (Figure 3a). Finally, ATG5 protein levels were reduced when treating WT or MLP-silenced cells with the PI3K inhibitor wortmannin (Figure 3a).

We next monitored autophagy induction by detection of the modification of endogenous LC3. LC3 exists in two forms: the free mature form LC3-I and the membrane-bound lipidated LC3-II form, which is a marker of autophagosome formation.^27^ We have observed decreased conversion of LC3-I to LC3-II in MLP-silenced myoblasts. Interestingly, starvation, a stimulus that activates autophagy, did not induce LC3-II accumulation in MLP-silenced myoblasts (Figure 3b, left panel). Similar results were obtained in myotubes. In fact, myotubes from control cells showed an increase in LC3-II that was, however, absent in MLP-silenced cells (Figure 3b). In myotubes starvation, however, increased LC3-II levels in both control and MLP-silenced cells (Figure 3b, right panel). LC3 accumulation during differentiation is shown in Supplementary Figure S1. In this case, LC3-II increase was observed in control cells after 48 h of differentiation, whereas no LC3-II accumulation was observed in MLP-silenced cells. A significant decrease in the number of LC3-II dots in MLP-silenced cells was also documented by immunofluorescence as shown in Figure 3c. Interpretation of LC3-II levels is subject to limitations since LC3-II is both produced and degraded during autophagy (autophagic flux). Therefore, the reduced LC3-II accumulation in MLP-silenced C2C12 muscle cells could be caused by either defective autophagy induction or excessive vesicle exhaustion. To circumvent this, it has been proposed that LC3-II levels should only be evaluated in the presence or absence of inhibitors, which block autophagosome-lysosome interaction and hence LC3-II degradation.^28^ For correct autophagy measurement, we have treated our cells with ammonium chloride (NH_4_Cl), which inhibits lysosome acidification thus resulting in the accumulation of LC3-II.^29^ Our results show that LC3-II levels in ammonium chloride-treated WT control cells markedly increased with respect to MLP-silenced cells where almost no increase was observed (Figure 3d). Moreover, inhibition of LC3-II increase was observed in both myoblasts and myotubes of MLP-silenced cells (Figure 3d). Supplementary Figure S2 shows that sustained (17 h) NH_4_Cl treatment resulted in an increased cell damage in myotubes from both WT and MLP-silenced cells confirming the important role of a correct autophagy for muscle cell differentiation.

It is known that autophagosome formation requires class III PI3K kinase activity. One of the most commonly used pharmacological approaches to inhibit autophagy *in vitro* involves the use of PI3 kinase inhibitors such as wortmannin or 3MA.^30^ Supplementary Figure S3 shows that treatment with early autophagy inhibitor 3-methyladenine (3MA) inhibited LC3-II accumulation in both WT control and MLP-silenced cells. Altogether, these data show an impairment of autophagy induction and LC3-II processing in MLP-silenced cells.

### MLP is indispensable for fasting-induced degradation of long-lived proteins

**Figure 4:**
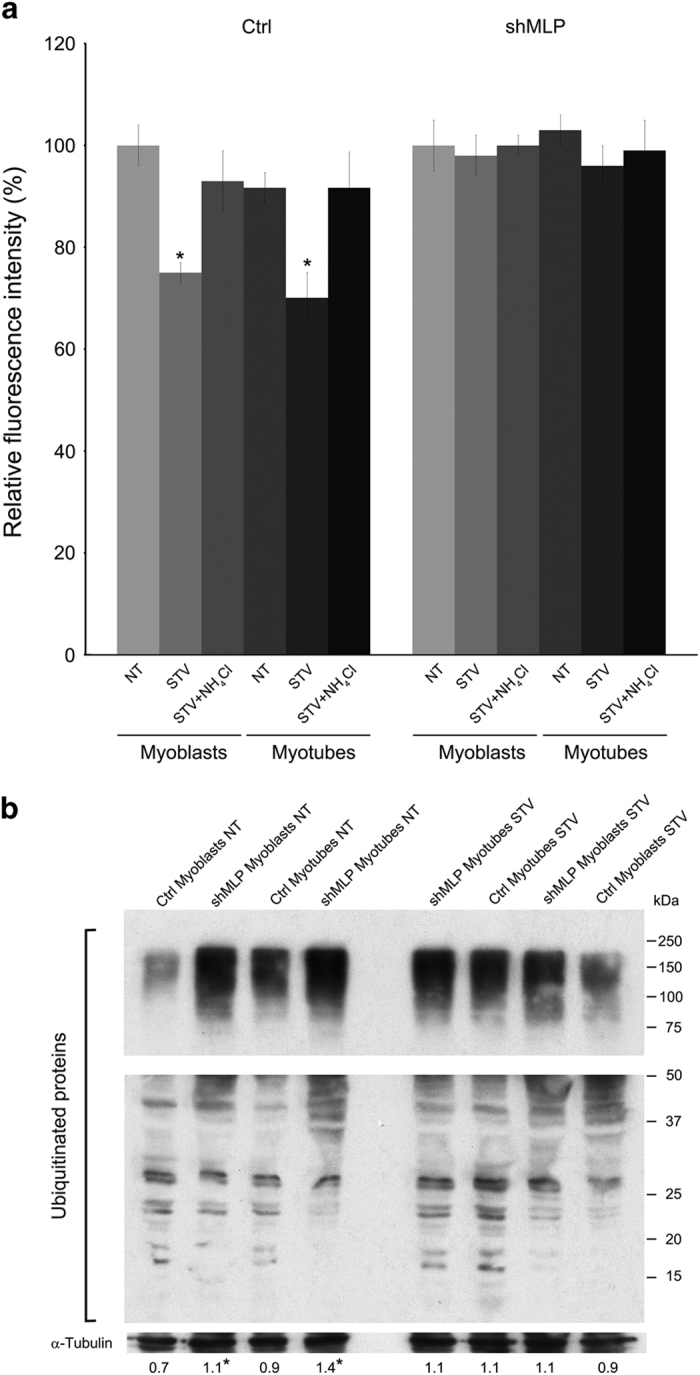
MLP silencing prevents degradation of long-lived proteins. (**a**) C2C12 control and shMLP myoblasts or myotubes cells were placed in a 96-well plate, labeled with AHA and then cultured in complete medium for 3 h. Alternatively, cells were cultured in starvation medium in the presence or absence of 10 mM NH_4_Cl for 3 h. Cells were then fixed, permeabilized and stained for 2 h with alkyne-Alexa Fluor 488 as described under Materials and Methods. Fluorescence from labeled long-lived proteins was measured using GloMax-multi detection system (Promega). The fluorescence intensity of the assay buffer was subtracted from each experimental sample. *Significantly different from control (ctrl) cells. Significance was set at *P*<0.05. (**b**) Ctrl and shMLP myoblasts and myotubes were processed to obtain whole protein lysates as described under Materials and Methods. Global protein ubiquitination was determined by western blot using a specific anti-ubiquitin antibody. *α*-Tubulin was used as loading control. Image in the figure is from the same membrane but has been separated in two parts due to different exposure times in order to show high and mid-low molecular weight proteins. Densitometric analysis was determined with ImageJ and average values from three separate experiments are reported below each band. *Significantly different from ctrl myoblasts; ^#^Significance was set at *P*<0.05.

Next, we used myoblasts and myotubes to investigate the effect of MLP silencing on protein turnover. During autophagy, cellular components, including protein aggregates, damaged or mutated proteins are degraded via a lysosome-dependent process to sustain metabolic homeostasis during nutrient deprivation. The rate of protein degradation measurement of long-lived proteins by AHA is an elegant method for the quantification of autophagic flux because it provides a precise numerical readout that reflects the fate of all long-lived cellular proteins and avoids the drawbacks associated with measuring a single autophagic substrate.^31^ When AHA is added to cultured cells, it is incorporated into proteins during protein synthesis. Induction of autophagy by starvation results in a significant reduction of the fluorescent intensity, consistent with other autophagic markers. For this experiment, we also stably overexpressed MLP in C2C12 cells (MLP^+^). Supplementary Figure S4A shows that MLP overexpression did not result in an increased MHC expression and differentiation of C2C12 cells. To assess the changes of AHA fluorescence intensity, both control WT, MLP-silenced and MLP-overexpressing myoblasts were stimulated by starvation for 3 h to induce autophagy. As shown in Figure 4a and in Supplementary Figure S4B, starvation caused a 20–30% significant reduction in the AHA signal intensity in WT and MLP-overexpressing C2C12 cell line. By contrast, degradation of long-lived protein was not induced by nutrient deprivation in MLP-silenced C2C12 cells indicating that autophagy process is impaired in these cells suggesting that MLP plays an important role in the turnover of long-lived protein (Figure 4a). We also verified our result by treating starved cells with ammonium chloride to inhibit autophagy. In this case, NH_4_Cl rescued AHA-decreased fluorescence in WT and MLP-overexpressing cells indicating the presence of a proper autophagic process (Figure 4 and Supplementary Figure S4B). Interestingly, in MLP-silenced cells, impaired autophagic process resulted in the accumulation of ubiquitinated proteins in both myoblasts and myotubes as shown in Figure 4b suggesting that autophagy dysfunction might contribute to the accumulation of protein aggregates.

### MLP-LC3-II co-localization

**Figure 5:**
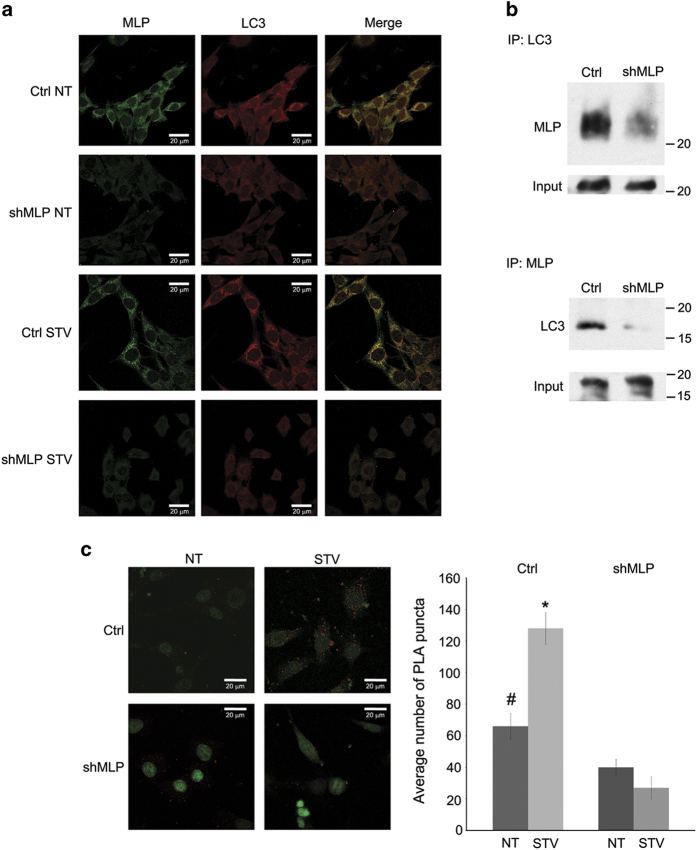
Co-localization of MLP and LC3 in cells cultured in basal and starvation condition. (**a**) Myoblasts were maintained under basal medium (NT) and serum-free starvation medium (STV) for 12 h. Co-localization of LC3 (red fluorescence) and MLP (green fluorescence) was studied by immunofluorescence as described under Materials and Methods. Yellow fluorescence represents areas of MLP and LC3 co-localization. Size bars are indicated in the figure. (**b**) Upper panel: WT control and MLP^+/−^ myoblast cells were processed to obtain whole cell extracts. Cellular extracts were immunoprecipitated with either an anti-LC3-II antibody or anti-rabbit IgG, electrophoresed on a SDS-PAGE gel and immunoblotted with anti-MLP antibody as described under Materials and Methods. Lower panel: cellular extracts were also immunoprecipitated with either MLP antibody or anti-goat IgG, electrophoresed on an SDS-PAGE gel and immunoblotted with anti-LC3 antibody. (**c**) Myoblast cells were maintained under basal medium (NT) and serum-free starvation medium (STV) for 12 h. LC3-MLP interaction was determined by PLA assay as described under Materials and Methods. PLA puncta were visualized by confocal microscope (left side). Size bars are indicated in the figure. The average number of PLA puncta in NT and STV-treated ctrl and shMLP cells was determined by ImageJ (NIH) as described under Materials and Methods. *Significantly different from control (ctrl) and shMLP starved cells. ^#^Significantly different from NT shMLP cells. Significance was set at *P*<0.05.

During autophagy, proteins destined for degradation are entrapped within autophagosome. Considering that both MLP and LC3 are microtubule-associated proteins (MAPs), we speculated that, also MLP could participate in autophagosome formation thereby colocalizing with other autophagosomal markers such as LC3. To test this hypothesis, C2C12 myoblasts were cultured in the presence of complete medium and serum-free starvation medium overnight and then subjected to immunofluorescence using anti-MLP and anti-LC3 antibodies. Our immunofluorescence localization studies revealed that, compared to untreated (NT) cells (Figure 5a), starvation induced a co-localization of endogenous MLP and LC3 on the formation of autophagosomes in WT myoblasts (Figure 5a). By contrast, in MLP-silenced myoblasts starvation was accompanied by a reduction of LC3-positive dots and a alteration of cellular morphology (Figure 5a).

We next used co-immunoprecipitation assay to determine whether MLP and LC3 are bound together in a complex in cells. For this purpose, whole cellular extracts from MLP wild-type and MLP-silenced myoblasts were first immunoprecipitated with an anti-LC3 antibody and then the amount of MLP revealed by western blot (Figure 5b, upper panel). We also did reverse immunoprecipitation (Figure 5b, lower panel). In both cases, the amount of LC3 that co-immunoprecipitated with MLP is reduced in MLP-silencing cells compared to WT control cells. These results suggest that MLP helps in the formation of autophagosome by interacting with LC3 protein. Immunofluorescence and immunoprecipitation results were further confirmed by proximity ligation assay (PLA). As shown in Figure 5c left panel, MLP associates with LC3 in WT cells under native conditions. Moreover, such association increased when autophagy was induced by starvation (Figure 5c). By contrast, no association or starvation induction was observed in MLP-silenced cells (Figure 5c). Quantification of PLA puncta revealed a significant increase of MLP-LC3 association in WT cells compared to MLP-silenced cells and also that such association increased when autophagy was induced by starvation (Figure 5c, right panel).

### Reduced expression of MLP results in the formation of membranous vesicles in MLP-silenced myofibers

**Figure 6:**
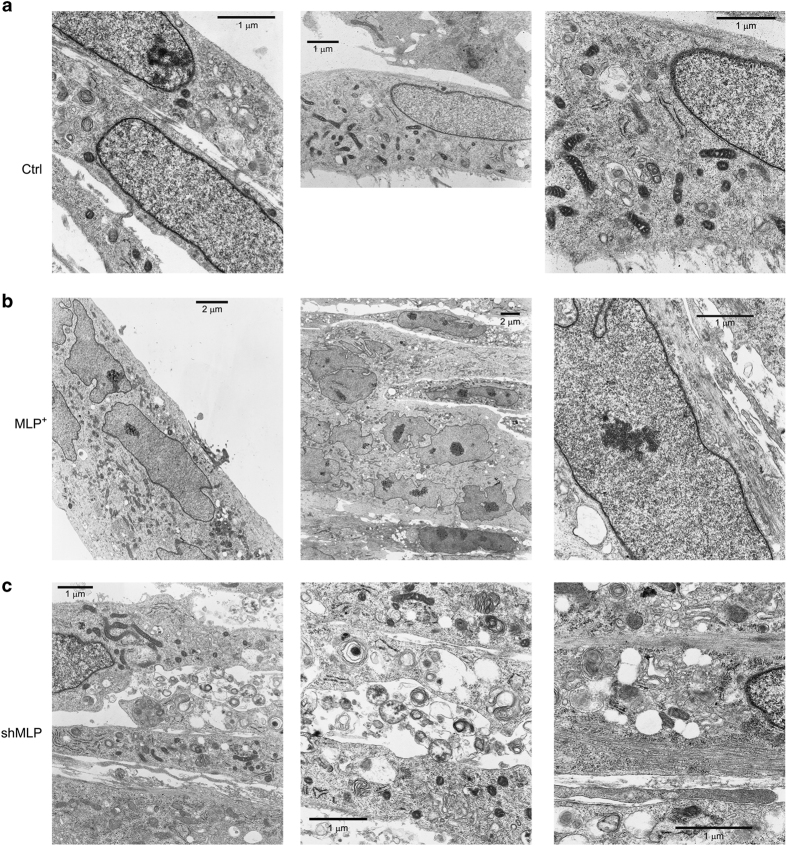
Morphological changes in C2C12 myotubes of shMLP myotubes. Myotubes from ctrl, MLP-overexpressing and shMLP cells were processed for transmission electron microscopy as described under Materials and Methods. (**a**) Normal myotube formation in wild-type cells with small autophagosome-like vacuoles and normal mitochondria (magnification from left to right: ×15 500, ×6600 and ×15 500). (**b**) Multinucleated myotubes formation in MLP-overexpressing (MLP^+^) cells with autophagosomes vacuoles and mitochondria resembling those seen in ctrl cells (magnification from left to right: ×2800, ×3800 and ×15 500). (**c**) Electron micrographs of shMLP myotubes showing a large number of concentric membranous structures with dense material and dappled dense bodies inside them as well as swollen and degenerating mitochondria (magnification from left to right: ×8900, ×15 500 and ×21 000). Size bars are indicated in the figure.

In general, autophagosomes quickly degrade after fusing with lysosome. If degradation proceeds too far, it is not easy to conclude whether those are autophagosome or membranous vacuoles. To confirm our results, we have performed transmission electron microscopy (TEM) analysis. As shown in the Figures 6a and b, both WT and MLP-overexpressing myotubes have several autophagosomes with normal structures whereas, MLP-silenced myotubes accumulates a large number of multimembranous structures resembling myelinic cellular structures that cannot be digested (Figure 6c). After considering our results on LC3-II expression and localization as well as results obtained after using autolysosome inhibitors, we suggest that the multimembranous structures that are seen in MLP-silenced myotubes are not real autophagosomes (Figure 6c). Of note, it has been shown that Atg5^−/−^ and Atg7^−/−^ mice muscle has same atrophic myofibers characterized with the accumulation of membranous inclusion bodies due to lack of autophagy.^10,32^ Interestingly, myoblasts from MLP-silenced cells accumulates large empty vacuoles that are not seen in WT or MLP-overexpressing myoblasts (Supplementary Figure S5). Of note, similar empty vacuoles have also been described when silencing p62/SQSTM1.^33^ Therefore, we speculate that MLP plays an important role in the autophagic process, which then facilitates differentiation of myoblasts into myotubes. In fact, we have seen reduced LC3-II during differentiation of MLP-silenced myoblasts (Figure 3). These results suggest an important role of MLP in the autophagic turnover of organelles with its defect resulting in the accumulation of abnormal organelles.

### Decreased MLP expression results in increased susceptibility of both myoblasts and myotubes to apoptosis

**Figure 7:**
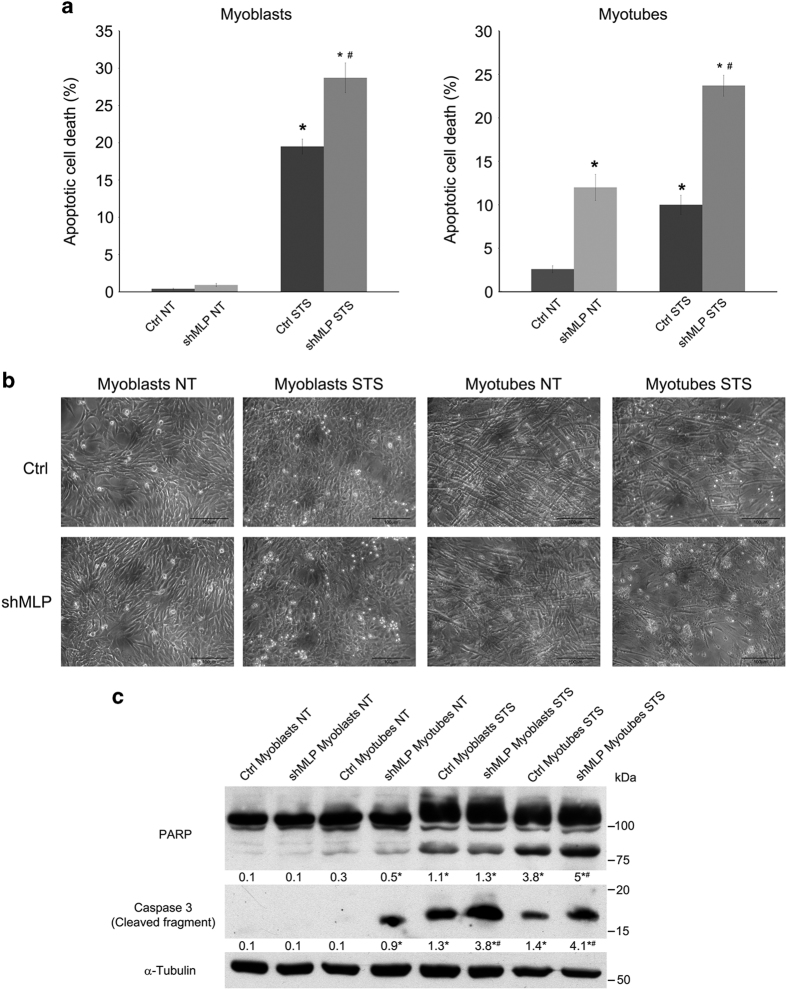
MLP downregulation confers susceptibility to apoptotic cell death. (**a**) Ctrl and shMLP myoblasts and myotubes were either left untreated (NT) or treated with 0.5 *μ*M STS for 16 h. Cells were then processed as described under Materials and Methods and cell death measured by PI exclusion by flow cytometry. *Significantly different from NT cells. ^#^Significantly different from ctrl STS-treated cells. Significance was set at *P*<0.05. (**b**) Ctrl and shMLP myoblasts and myotubes were plated on a 100-mm dish, either left untreated (NT) or treated with STS 0.5 *μ*M for 16 h. Pictures of the cells were taken at ×20 with a digital camera mounted on an EclipseNet 2000 microscope. (**c**) Ctrl and shMLP myoblasts and myotubes were either left untreated (NT) or treated with 0.5 *μ*M STS for 16 h. Cells were then processed for western blot analysis as described under Materials and Methods. Cleavage of apoptotic markers PARP-1 and caspase-3 was monitored in MLP-silencing cells compared to wild-type cells. *α*-Tubulin was used as loading control. Densitometric analysis was determined with ImageJ and average values from three separate experiments are reported below each band. *Significantly different from ctrl or shMLP myoblasts; ^#^significantly different from ctrl STS-treated myoblasts or myotubes. Significance was set at *P*<0.05.

Recently, it has been shown that autophagy protects myoblasts against apoptosis during differentiation.^34^ Considering our results showing a role for MLP in autophagy, we intended to determine the role of MLP under apoptotic stress. For this purpose, we decided to evaluate the survival response of C2C12 myoblasts and myotubes to cell death stimuli following MLP downregulation. Therefore, we have treated both control WT and MLP-silenced C2C12 cells with 0.5 *μ*M staurosporine (STS) for 16 h. We have quantified cell death by measuring both the sub-G1 cell population of propidium iodide (PI)-positive cells by flow cytometry. In the presence of STS, we have seen increased resistance of WT cells with a percentage of cell killing of 20 and 10% in myoblasts and myotubes, respectively (Figure 7a). However, in MLP-silenced cells such resistance is decreased with a percentage of cell killing of 28 and 23% in myoblasts and myotubes, respectively (Figure 7a). Interestingly, differentiation of MLP-silenced cells also significantly increased the percentage of apoptosis compared to WT cells (Figure 7a, right panel). The increased number of dead cells in MLP-silenced myoblasts and myotubes after STS treatment (measured in Figure 7a) is shown in Figure 7b. Again, no difference in the percentage of cell death was observed between WT and MLP-overexpressing cells (Supplementary Figure S6). Furthermore, to validate our flow cytometric results, we have also analyzed cleaved caspase-3 and PARP-1, markers of intrinsic apoptosis, in the presence of STS by western blot. Figure 7c shows increased cleavage of PARP-1 and caspase-3 in MLP-silenced cells relative to control. Moreover, in MLP-silenced cells, we observed caspase-3 cleavage also during differentiation (Figure 7c). Collectively, these results show an increase in apoptosis in the absence of MLP, an effect probably due to the fact that autophagy is impaired in MLP-silenced cells.

## Discussion

Although MLP has been suggested to act as a mechanical stress sensor^35^ in the downstream signaling cascades, apoptotic or autophagy role by MLP has not yet been determined. This study addresses several novel aspects of MLP regulation of autophagy in skeletal muscles.

In the present study, we generated MLP-silenced skeletal myoblast C2C12 to analyze its role in autophagy. The major finding is that endogenous MLP regulates basal autophagy and in response to acute nutrient starvation. C2C12 myoblasts were chosen as a cell line model because MLP is expressed only in muscle cells and cardiomyocytes. In these cells, autophagy was measured by using four methods: LC3-II lipidation assay; measurement of autophagy-related gene expression; a long-lived protein degradation assay; and visualization of MLP co-localization with LC3 using confocal immunofluorescence, immunoprecipitation and PLA. In addition, we have also determined the functional role of MLP in myoblasts and myotubes under apoptotic stress. Initially, we monitored the expression of MLP during differentiation of myoblasts showing that knockdown of MLP in C2C12 myoblasts results in the inhibition of myotube formation. We have shown reduced expression of MLP results in the downregulation of genes including MyoD1 and myogenin as previously reported.^36,37^ We assume that MLP might have a role in myotube formation via regulation of cellular regulatory pathways such as autophagy regulatory system. Autophagy is a bulk degradation process in which eukaryotes maintain cellular remodeling and structural integrity under nutrient rich or poor condition.^15,38^ Our results indicate that MLP plays a beneficial role in muscle homeostasis via basal autophagy. In our model, antisense downregulation of MLP in cultured skeletal myoblasts and myotubes prevented the transcription of some autophagy-related genes under basal level suggesting a critical role of MLP in the induction of autophagy (Figure 3). After starvation, we have also seen increased induction of those genes in the presence of endogenous MLP in WT control cells compared to MLP-silenced myoblasts. The formation of autophagosomes was confirmed by using the autophagy marker LC3-II. The reduction of LC3-II is accompanied with enhanced presence of damaged materials and organelles.

After treatment with pharmacological inhibitors such as ammonium chloride, we have seen increased accumulation of LC3-II in wild-type control cells compared to MLP-silenced cells. Moreover, when autophagy is blocked at the early stages of autophagy using 3MA, autophagic vacuole formation is inhibited, characterized with reduced LC3-II synthesis (Supplementary Figure S3).

The formation of autophagosomes was confirmed by using immunostaining of autophagy marker LC3. Immunofluorescence analysis revealed that LC3-positive dots appeared in WT control cells but did not form cup-shaped and ring-like structures in MLP-silenced myoblasts (Figure 3c). It seems to us that the modification and levels of LC3 molecules under starvation were affected in the mutant cells. However, mechanisms responsible for this relationship are not entirely clear. Induction of autophagy-related gene is not likely to be the only important factor in determining autophagy system. Other important factors such as post-translational modification, protein-protein interactions of autophagy-related proteins also regulate initiation of autophagy. Autophagy is regulated at several steps involving complex interactions between multiple signaling pathways. A double immunofluorescence study confirmed a co-localization of the autophagic marker LC3 and the MLP (Figure 5a). Reduced expression of MLP in MLP-silenced cells showed decreased band intensity between these two proteins in co-immunoprecipitation assay (Figure 5b). Finally, we confirmed MLP and LC3 interaction by PLA demonstrating a close proximity of these two proteins that increases under starvation and is abrogated when MLP expression is silenced (Figure 5c). The co-localization and co-immunoprecipitation of LC3 and MLP observed in WT control cells suggest the presence of an interaction between these two proteins. In particular, we speculate that the LIM domain of MLP interacts with LC3 during the autophagy process and thus facilitates the autophagy process. This interaction would be important for cellular remodeling occurring during differentiation or starvation.

In muscle, the persistent activation of catabolic pathways results in muscle atrophy and weakness.^39^ In our model, we have observed multiple abnormalities of organelles (e.g., the presence of concentric membranous structure and accumulation of deformed mitochondria) in MLP-deficient myotubes (Figure 6c). The morphological features of myotube by light microscopy pointed us to observe abnormal myofiber regions of varying size and shape, harboring vacuolated muscle fibers and abnormal membranous material within or lining the vacuoles in MLP-silenced myotubes. It might be that those membranous structures that incorporate intracellular materials result in myofiber disorganization. Our results suggest that lack of MLP results in impaired autophagy, which then causes in the accumulation of membranous vacuoles during myotube formation and thus generates degenerated myofiber formation. We have also observed accumulation of dysfunctional mitochondria in MLP-silenced myotubes. It has been determined that dysfunctional mitochondria contribute to the pathogenesis of Ullrich and Bethlem dystrophies.^40–42^ Recently, it has been shown that mutation of another LIM domain protein named four-and-half LIM domain protein 1 (FHL1) results in chronic myopathies characterized with increased accumulation of aggregated proteins and vacuoles with misfolded proteins.^43^

Moreover, autophagy has been shown to be important in the normal maintenance of muscle mass by deletion of tissue-specific genes essential for autophagy (e.g., Atg7) and revealed muscle degeneration with the accumulation of damaged mitochondria, protein aggregates and polyubiquitinated proteins.^10^ In particular, autophagy is required for the clearance of this ubiquitin-positive protein aggregates.^44^ We have addressed this issue in MLP-mutant skeletal myoblasts and myotubes to determine the interplay between autophagy and protein accumulation. Interestingly, C2C12 MLP-silenced myoblasts and myotubes showed increased ubiquitin-positive aggregates compared with wild-type control cells indicating that there is a failure of autophagosomal–lysosomal fusion and a functional deficiency of autophagy (Figure 4b). Of note, neural-specific ablation of atg5 or atg7 leads to accumulation of ubiquitin-positive protein aggregates and progressive loss of neurons in mice proving that autophagy is required for the clearance of protein aggregates and protects individuals from neurodegeneration.^45,46^ Moreover, it has been determined that suppression of autophagy in skeletal muscle results in the accumulation of toxic ubiquitinated proteins and it has remarkable role in muscle damage in Pompe disease.^47^

Our results suggest that basal level of autophagy in some extent may occur in the presence of MLP and certain level of autophagy is required to preserve the myotube structure and function during myotube formation and thereby prevents alteration of myotube morphology or integrity. The importance of basal autophagy is highlighted in Danon disease, in which cardioskeletal myopathy arises due to deficiency of LAMP2, a lysosomal membrane protein. In the absence of LAMP2, fusion of autophagosomes with lysosomes is blocked, leading to accumulation of long-lived proteins and consequent myopathy.

Autophagy is characterized by the degradation of long-lived protein.^48^ Although, autophagy can be induced by starvation, this pathway may take place even in the presence of feeding condition or in complete cell medium at basal level. This constitutive pathway may be important for turnover of organelles and cytoplasmic proteins. In our study, we have shown that reduction of MLP results in impaired autophagy in skeletal myoblasts and myotubes. During starvation condition, cells digest long-lived proteins to release amino acids. In our case, autophagy-deficient MLP-silenced cells display altered degradation of long-lived proteins after 3 h of starvation (Figure 4a).

Autophagy is a recycling process of cytoplasmic components, such as long-lived proteins and damaged organelles. In several diseases, there is accumulation of protein aggregates named huntingtin, parkin and beta amyloid plaque in Huntington’s disease, Parkinson’s disease and Alzheimer’s disease, respectively due to lack of autophagy. Mutant mice with neuron-specific deletion of Atg5 or Atg7 results in the development of neurodegeneration and cytoplasmic inclusion bodies that contain protein aggregates.^45,46^

Inhibition of autophagy results in the accumulation of damaged waste materials inside the cell and increases the promotion of apoptosis.^49^ When autophagy is insufficient in cardiac-specific Atg5-deficient mice, apoptosis is promoted, leading to cell death.^49^ The phenotype of increased death of mutant myoblasts and myotubes is determined in the presence of STS and compared with that of wild-type control. The contribution of MLP in apoptosis is confirmed by RNA interference approaches, showing that MLP downregulation markedly sensitized cells to STS-induced cell death. In the case of MLP-deficient myoblasts and myotubes, it seems that reduced autophagy might facilitate enhancement of apoptotic cell death during STS treatment (Figure 7). Autophagy can protect cells by preventing them from undergoing apoptosis.^50^ It is not still clear how autophagy stops cells from undergoing apoptosis; one suggested mechanism is that autophagy helps in the sequestration of damaged mitochondria,^50^ thus, preventing released cytochrome *c* from being able to form a functional apoptosome in the cytoplasm. A failure in autophagy induces accumulation of death-signaling components, which triggers apoptosis and myofiber degeneration.

In conclusion, our findings point to a causative role of MLP in autophagy activation, and suggest that lack of MLP impairs autophagy in skeletal muscle causing a defective removal of organelle or damaged protein, which then induces profound muscle loss and weakness. Thus, we suggest that MLP might have dual function such as cytoarchitecture as well as signal transduction in the autophagy process. Future studies of the autophagic flux in muscle cells from patients and animal models for MLP deficiency are needed in order to understand in detail how and to which extent deregulation of autophagy contributes to the pathogenesis of MLP-deficient myopathies.

## Materials and Methods

### Cell cultures

The C2C12 mouse myoblast cell line was purchased from LGC Standards (Milan, Italy) and maintained in DMEM medium (Sigma-Aldrich, St. Louis, MO, USA) containing 100 units/ml penicillin, 0.1 mg/ml streptomycin and 15% heat-inactivated fetal bovine serum (FBS) in the presence of temperature at 37 °C in a humidified atmosphere of 5% CO_2_ and 95% air. Differentiation into myotubes was achieved by culturing C2C12 myoblasts in complete medium and then switching into DMEM medium supplemented with 1% FBS when cells were 90% confluent. Differentiation was maintained for the time indicated in the figures.

### Generation of MLP overexpressing and silenced transfectants

C2C12 cells were stably transfected with a pcDNA3.1 expression vector encoding for mouse CSRP3/MLP-Flag (OriGene, Rockville, MD, USA) for overexpression and for mouse MLP shRNA (Sigma-Aldrich) for silencing. Stable clones were generated using FuGENE transfecting chemical reagent according to the manufacturer’s instructions (Promega, Milan, Italy). Briefly, 10 000 cells were seeded on a six-well plate. The following day the cells were transfected with FuGENE reagents. At first, FuGENE is mixed with serum-free DMEM media at room temperature for 5 min. Subsequently, plasmid containing MLP-silencing shRNA, overexpressing plasmid and scramble was added to the FuGENE-containing media and incubated at room temperature for 30 min. Selection of stable clones was started 48 h later with the addition of 3 *μ*g/ml of puromycin and G418 (Sigma-Aldrich) for silenced and overexpressing clones, respectively. Puromycin-resistant and G418-resistant cells were selected, pooled and examined by western blot and quantitative qRT-PCR analysis for CSRP3 expression and used for further analysis.

### Treatment protocols and antibodies

For serum starvation, cells were washed with phosphate-buffered saline (PBS) and then cultured in serum-free DMEM for 17 h. To block autophagy, cells were treated with ammonium chloride (10 mM) or with 3MA (5 mM) or with wortmannin (400 nM). All these molecules were from Sigma-Aldrich. The following primary antibodies were used: goat anti-CSRP3/MLP (Novus Biological, Abingdon, UK), mouse anti-MHC (Novus Biological), rabbit anti-ATG5 (MBL, Woburn, MA, USA), rabbit anti-ATG7 (Novus Biological), mouse anti-ubiquitin (Sigma-Aldrich), mouse anti-Flag (Sigma-Aldrich), rabbit anti-LC3B (Novus Biological), rabbit anti-PARP-1 antibody (Santa Cruz Biotechnology Inc., Heidelberg, Germany), mouse anti-tubulin (Santa Cruz Biotechnology Inc.) and anti-caspase-3 (Santa Cruz Biotechnology Inc.). The following secondary antibodies were used: mouse anti-rabbit Alexa 488 (Molecular Probes, Eugene, Oregon, USA), goat anti-rabbit Alexa 594 (Molecular Probes), mouse anti-rabbit horseradish peroxidase (HRP), goat anti-mouse HRP (Amersham Biosciences, Piscataway, NJ, USA) and donkey anti-goat HRP (Santa Cruz Biotechnology Inc.).

### Microscopy

Cellular morphology was evaluated in proliferating myoblasts and differentiated myotubes by phase-contrast microscopy without preliminary fixation. Pictures were produced using an inverted microscope (Nikon Eclipse TE2000U, Florence, Italy) and a digital camera (Nikon DS5Mc).

### RNA isolation and real-time quantitative PCR

Cells were plated in 100-mm dishes in complete DMEM medium. Following proliferation for 24 h, TRIzol (Life Technologies, Monza, Italy) was added in each dish, scraped the cells and collected in 1.5-ml Eppendorf tube, and then vortexed vigorously for 10 s. Afterwards, RNA extraction was carried out according to the protocol as indicated by the supplier. Approximately, 2 *μ*g of RNA was reverse transcribed by using Invitrogen SuperScript III first-strand synthesis kit according to manufacturer’s instructions. Approximately, 10 ng of cDNA aliquots were subjected to real-time PCR with SYBR select master mix (Applied Biosystems, Foster City, California, USA). All PCR reactions were performed in triplicate. PCR amplifications were performed as follows: 50 °C for 10 min and 40 cycles each with 95 °C for 15 s and 60 °C for 1 min using an ABI PRISM 7900 sequence detector (Applied Biosystems). Amplification data were analyzed using the Sequence Detector version 1.7 software (Applied Biosystems). Statistical analysis of real-time PCR results were performed using mean normalized cycle threshold (ΔC_t_). Primer sets for all amplicons were designed using the Roche Primer Design Centre (Roche, Milan, Italy). Primers used are as follows: ATG5 forward: 5′-AGCAGCTCTGGATGGGACTGC-3′, ATG5 reverse: 5′-GCCGCTCCGTCGTGGTCTGA-3′; ATG7 forward: 5′-GCTCCTCATCACTTTTTGCCAACA-3′, ATG7 reverse: 5′-GGAGCCACCACATCATTGC-3′; MYOD1 forward: 5′-AGCACTACAGTGGCGACTCA-3′, MYOD1 reverse: 5′-GGCCGCTGTAATCCATCA-3′; MYOG1 forward: 5′-ACTCCCTTACGTCCATCGTG-3′, MYOG1 reverse: 5′-CAGGACAGCCCCACTTAAAA-3′; GAPDH forward: 5′-TGCCAAGTATGATGAACATCAAGAAG-3′, GAPDH reverse: 5′-GGTCCTCAGTGTAGCCCAAGAT-3′.

### Protein extraction and western blot assay

Cells (1×10^6^) for whole cell lysate were pelleted at 700×*g* (5 min at 4 °C) and lysed in 50 *μ*l of cell lysis buffer (20 mM Tris (pH 7.4), 100 mM NaCl, 1% Triton (Sigma-Aldrich), 1 mM phenylmethylsulfonyl fluoride, 10 *μ*g/ml leupeptin and 10 *μ*g/ml aprotinin). After 30 min on ice, the lysates were clarified by centrifugation (10 min at 4 °C) and the supernatant was collected. Protein concentration was determined by the Bradford assay (Bio-Rad, Milan, Italy). Equivalent amounts of protein were electrophoresed on SDS-PAGE gels. Kaleidoscope prestained standards (Bio-Rad) were used to determine molecular weight. The gels were then electroblotted onto PVDF membranes. After blocking with 5% milk, membranes were incubated with the primary antibody overnight. Finally, the relevant protein was visualized by staining with the appropriate secondary horseradish peroxidase-labeled antibody for 1 h followed by enhanced chemiluminescence. Densitometric analysis of the bands, relative to *α*-tubulin, was performed using ImageJ software (National Health Institute, Bethesda, MD, USA).

### Immunofluorescence and confocal microscopy

Cells were plated on glass cover slides in complete medium and incubated overnight at 37 °C and 5% CO_2_. Cells were then treated with starvation medium for autophagy stimulation. After that cells were washed with PBS and fixed in 4% paraformaldehyde for 10 min. The cells were then washed twice with PBS and permeabilized with 0.5% Triton X-100 for 6 min (Sigma-Aldrich). After a PBS wash, cells were incubated with primary antibody diluted in PBS-1% BSA at 4 °C. Cells were then washed three times for 5 min each with PBS. After primary antibody incubation and washing, cells were incubated in the dark in fluorescent secondary antibody for 1 h at room temperature and washed thrice with PBS. Coverslips were mounted on antifade mountant (ProLong Diamond Antifade Mountant, Life Technologies). Fluorescence intensity was visualized with an LSM 510 confocal microscope (Zeiss, Milan, Italy).

### Immunoprecipitation assay

Proteins were extracted as mentioned above. After determining protein concentration using the Bradford assay (Bio-Rad), 1 mg of lysate was brought to a final volume of 1 ml with lysis buffer including phosphatase inhibitor Lysates were precleared with 5 *μ*g of appropriate control IgG (Santa Cruz Biotechnology Inc., sc-2763) and 20 *μ*l of protein A/G plus-agarose (Santa Cruz Biotechnology Inc., sc-2003) and kept on a rotator for 1 h at 4 °C. Lysates were centrifuged (500×*g* for 5 min at 4 °C) and 5 *μ*g of MLP or LC3-II antibody or corresponding IgG was added to the precleared lysates and kept on ice for ~3–5 h. After incubation, 30 *μ*l of protein A/G plus-agarose was added to each tube and kept on a rotator overnight at 4 °C. Lysates were then centrifuged (500×*g* for 5 min at 4 °C). The pellet fractions were washed four times with PBS-PI and then resuspended in 20 *μ*l of loading buffer. Samples were electrophoresed on a 12% SDS-PAGE gel and immunoblotted with the appropriate antibody as indicated in the figures.

### Proximity ligation assay

Cells were plated on glass cover slides in complete medium and incubated overnight at 37 °C and 5% CO_2_. Cells were then treated with starvation medium for autophagy stimulation. Cells were then fixed and permeabilized as described above. After a PBS wash, cells were incubated with appropriate combination of rabbit anti-LC3 and goat anti-MLP primary antibodies diluted in PBS-1% BSA at 4 °C. PLAs were performed using a Duolink (Sigma-Aldrich) kit according to the manufacturer’s instructions.

Nuclei were stained with Sytox green (Life Technologies) 50 nm for 10 min. Coverslips were mounted on antifade mountant (ProLong Diamond Antifade Mountant, Life Technologies). Fluorescence intensity was visualized with an LSM 510 confocal microscope (Zeiss). Images were analyzed in ImageJ to calculate the density of PLA puncta.

### Autophagic long-lived proteolysis assessment by AHA

Autophagic proteolysis of long-lived proteins was determined by using Click-iT metabolic labeling for proteins (Life Technologies, C10428) as previously published.^31^ Briefly, cells with 70% confluency were cultured in a 96-well plate (for fluorimeter measurements). The day after the cells were washed in PBS (Sigma-Aldrich) and then cultured in L-methionine-free medium for 2 h to deplete intracellular methionine reserves. Cells were then pulsed for 18 h with 50 *μ*M Click-iT AHA (l-azidohomoalanine; Life Technologies) in methionine-free medium with 10% dialyzed FBS to eliminate the source of methionine. Afterwards, cells were washed with PBS+3% BSA (Sigma-Aldrich) and cultured for 2 h in complete medium containing methionine to chase out short-lived proteins. Cells then underwent various treatments as indicated in the figure legends. Starvation was achieved by incubating cells in amino acid-free medium supplemented with 0.1% BSA and 2 mM l-methionine.

Finally, cells were washed in PBS+3% BSA. The detection of click reaction was obtained by incubating fixed cells for 2 h with alkyne-Alexa Fluor 488 (Life Technologies) in Click-iT cell reaction buffer kit. The reaction mixture was removed by washing samples with PBS+3% BSA. Fluorescence from labeled long-lived proteins was measured using GloMax-multi detection system (Promega). The fluorescence intensity of the assay buffer was subtracted from each experimental sample.

### Transmission electron microscopy

C2C12 myoblasts or myotubes were detached from the dish using a manual scraper, washed a couple of times with PBS. The cells were then suspended and fixed overnight at 4 °C in 2% glutaraldehyde with 1% tannic acid in 0.1 M sodium cacodylate, pH 7.3. The cells were rinsed three times in the sodium cacodylate buffer and then incubated in 2% osmium tetroxide in the same buffer for 2 h at room temperature. Afterwards, the cells were rinsed three times in distilled water and exposed to 1% uranyl acetate in water for 15 min at room temperature. The cells were rinsed twice in distilled water, spun down into 3% agarose at 45 °C and cooled to form blocks. The agarose blocks were dehydrated in graded steps of acetone and embedded in Spurr’s low-viscosity media. Following polymerization overnight at 65 °C, 80-nm sections were cut on a Reichert-Jung Ultracut E ultramicrotome (Leica Microsystems, Milan, Italy) and picked up on copper grids. The grids were post-stained in uranyl acetate and bismuth subnitrate. The sections were observed on a Philips CM-10 TEM (FEI Italia, Milan, Italy) and micrographs were recorded on a Kodak 4489 sheet film.

### Apoptosis analysis

C2C12 myoblasts and myotubes were grown in 10-mm dishes. Apoptosis was induced by treating cells with 0.5 *μ*M STS. After the treatment, cells were harvested by centrifugation (10 min at 1600 r.p.m. at 4 °C), washed with 5 ml PBS and resuspended in 500 *μ*l PBS to which 5 ml of cold 70% EtOH was slowly added while stirring. After overnight incubation at 4 °C, cells were centrifuged at 1600 r.p.m. for 5 min at 4 °C and washed once with PBS. The cells were then resuspended in 500 *μ*l of a solution composed of 50 *μ*g/ml PI, 250 *μ*g/ml RNase A and PBS 1× and kept at 37 °C for 30 min.

### Statistical analysis

The results are expressed as means±S.D. and 95% confidence intervals (95% CIs). Before using parametric tests, the assumption of normality was verified using the Shapiro-Wilk W-test. Student's paired *t*-test was used to determine any significant differences before and after treatment. Significance was set at *P*≤0.05. SPSS statistical software package (SPSS Inc., version 13.0.1 for Windows, Chicago, IL, USA) was used for all statistical calculations.

## Abbreviations

MLP/CSRP3: muscle LIM protein/cysteine-rich protein 3
PLA: proximity ligation assay
MAP1LC3B: microtubule-associated protein 1 light chain 3 beta
Myog: myogenin
Myod1: myogenic differentiation 1
ATG: autophagy related
MHC: myosin heavy chain
PARP: poly(ADP-ribose)polymerase
PI: propidium iodide
PI3K: phosphoinositide-3-kinase
3MA: 3*-*Methyladenine
MAP: microtubule-associated protein
LAMP2: lysosomal-associated membrane protein 2.
