# Supplementary material for: Muscle LIM protein/CSRP3: a mechanosensor with a role in autophagy
Source: Cell Death Discov. 2015 Aug 3;1:15014–. doi: 10.1038/cddiscovery.2015.14 (PMC4981024; doi:10.1038/cddiscovery.2015.14)
Supplement: Supplementary figures and legends [file cddiscovery201514-s1.ppt]

## Slide 1
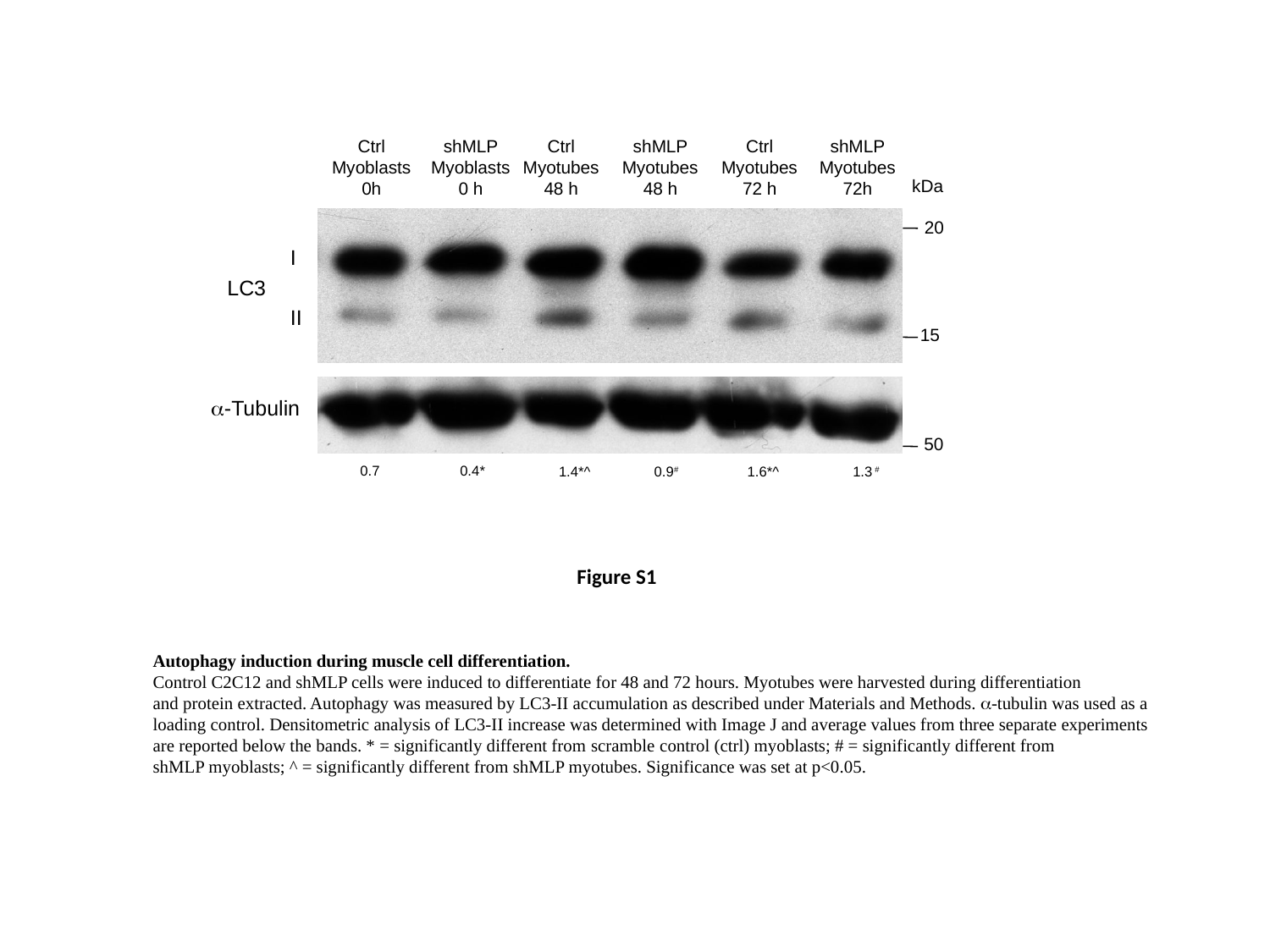

Ctrl
Myoblasts
0h
shMLP
Myoblasts
0 h
Ctrl
Myotubes
48 h
shMLP
Myotubes
48 h
Ctrl
Myotubes
72 h
shMLP
Myotubes
72h
kDa
20
I
LC3
II
 15
α-Tubulin
50
0.7
0.4*
1.4*^
0.9#
1.6*^
1.3 #
Figure S1
Autophagy induction during muscle cell differentiation.
Control C2C12 and shMLP cells were induced to differentiate for 48 and 72 hours. Myotubes were harvested during differentiation
and protein extracted. Autophagy was measured by LC3-II accumulation as described under Materials and Methods. α-tubulin was used as a
loading control. Densitometric analysis of LC3-II increase was determined with Image J and average values from three separate experiments
are reported below the bands. * = significantly different from scramble control (ctrl) myoblasts; # = significantly different from
shMLP myoblasts; ^ = significantly different from shMLP myotubes. Significance was set at p<0.05.

## Slide 2
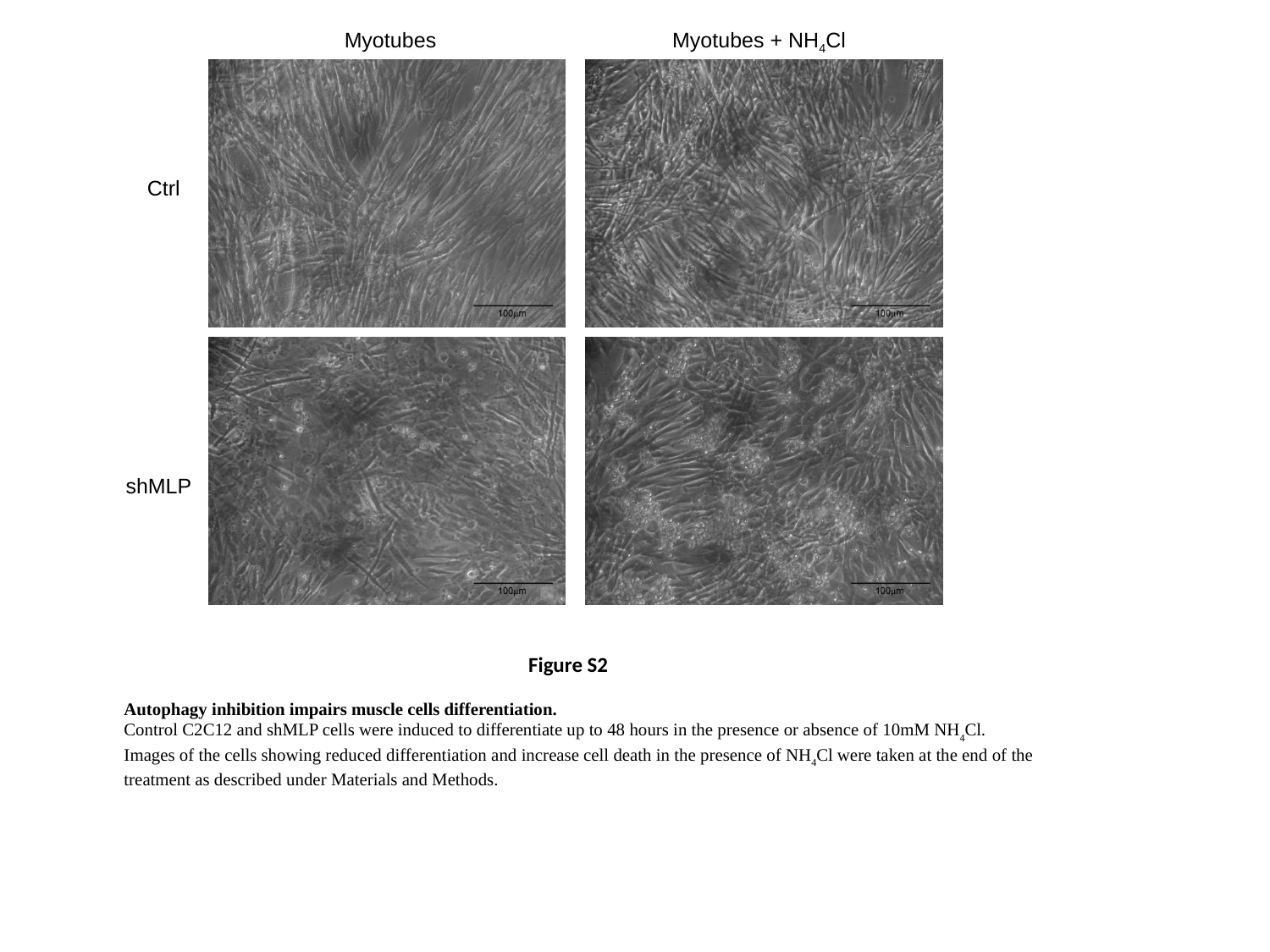

Myotubes
Myotubes + NH4Cl
Ctrl
shMLP
Figure S2
Autophagy inhibition impairs muscle cells differentiation.
Control C2C12 and shMLP cells were induced to differentiate up to 48 hours in the presence or absence of 10mM NH4Cl.
Images of the cells showing reduced differentiation and increase cell death in the presence of NH4Cl were taken at the end of the
treatment as described under Materials and Methods.

## Slide 3
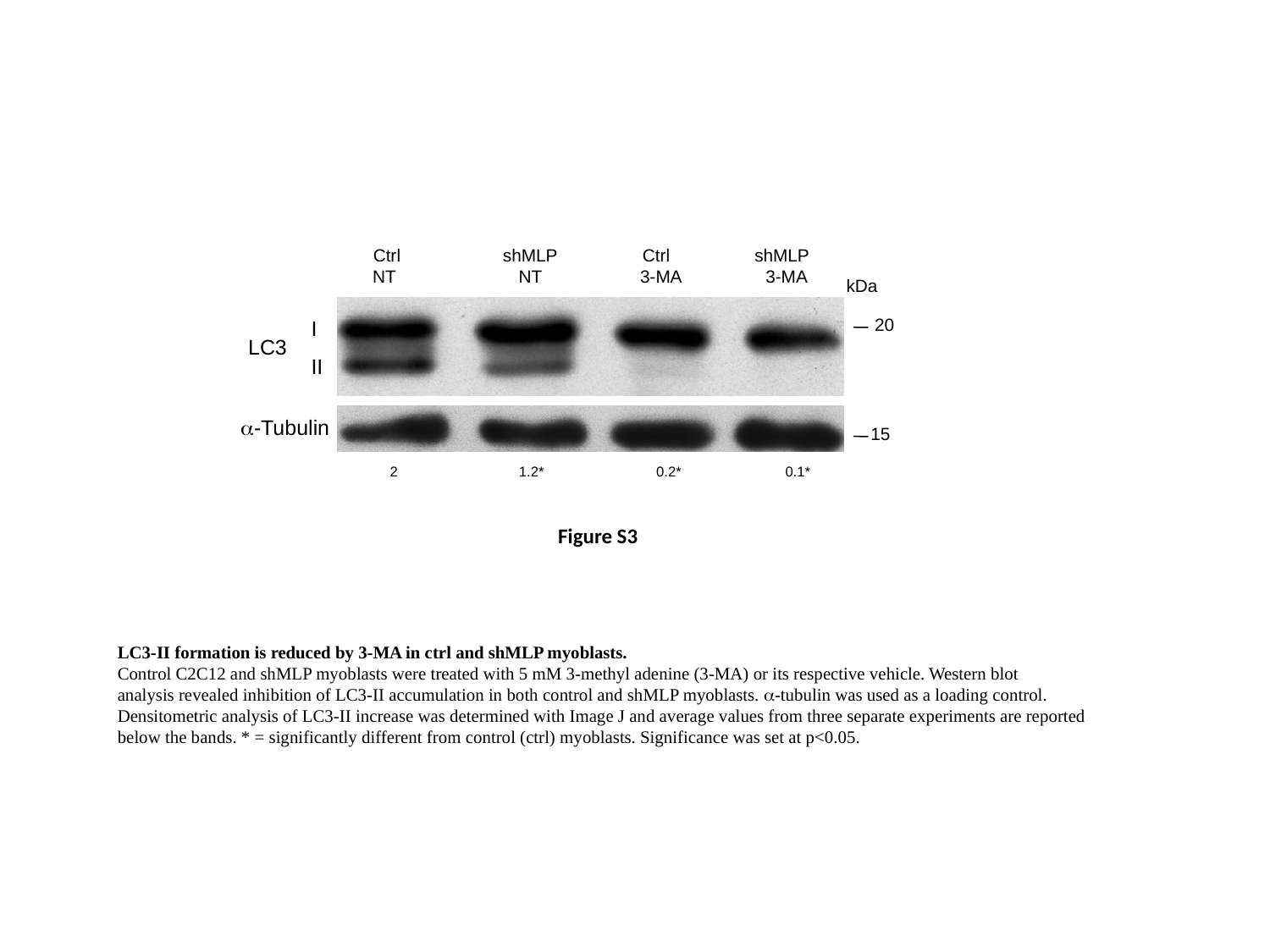

Ctrl
NT
 shMLP
 NT
 Ctrl
 3-MA
shMLP
 3-MA
kDa
20
I
LC3
II
α-Tubulin
 15
2
1.2*
0.2*
0.1*
Figure S3
LC3-II formation is reduced by 3-MA in ctrl and shMLP myoblasts.
Control C2C12 and shMLP myoblasts were treated with 5 mM 3-methyl adenine (3-MA) or its respective vehicle. Western blot
analysis revealed inhibition of LC3-II accumulation in both control and shMLP myoblasts. α-tubulin was used as a loading control.
Densitometric analysis of LC3-II increase was determined with Image J and average values from three separate experiments are reported
below the bands. * = significantly different from control (ctrl) myoblasts. Significance was set at p<0.05.

## Slide 4
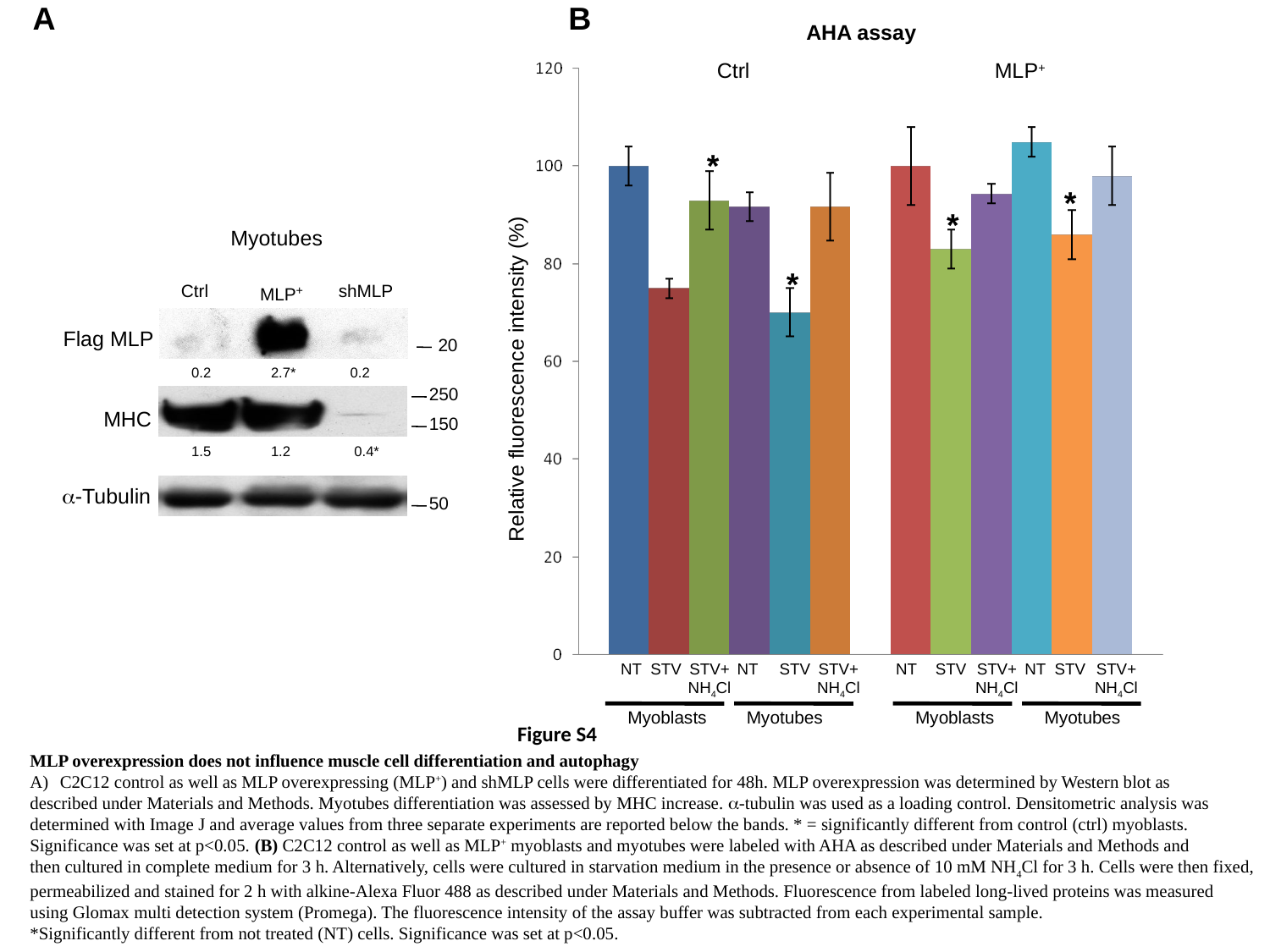

A
B
AHA assay
Ctrl
MLP+
*
*
*
Myotubes
*
Ctrl
 MLP+
shMLP
Flag MLP
20
0.2
2.7*
0.2
Relative fluorescence intensity (%)
250
 MHC
150
1.5
1.2
0.4*
α-Tubulin
50
NT
STV
STV+
NH4Cl
NT
STV
STV+
NH4Cl
NT
STV
STV+
NH4Cl
NT
STV
STV+
NH4Cl
Myoblasts
Myotubes
Myoblasts
Myotubes
Figure S4
MLP overexpression does not influence muscle cell differentiation and autophagy
C2C12 control as well as MLP overexpressing (MLP+) and shMLP cells were differentiated for 48h. MLP overexpression was determined by Western blot as
described under Materials and Methods. Myotubes differentiation was assessed by MHC increase. α-tubulin was used as a loading control. Densitometric analysis was
determined with Image J and average values from three separate experiments are reported below the bands. * = significantly different from control (ctrl) myoblasts.
Significance was set at p<0.05. (B) C2C12 control as well as MLP+ myoblasts and myotubes were labeled with AHA as described under Materials and Methods and
then cultured in complete medium for 3 h. Alternatively, cells were cultured in starvation medium in the presence or absence of 10 mM NH4Cl for 3 h. Cells were then fixed,
permeabilized and stained for 2 h with alkine-Alexa Fluor 488 as described under Materials and Methods. Fluorescence from labeled long-lived proteins was measured
using Glomax multi detection system (Promega). The fluorescence intensity of the assay buffer was subtracted from each experimental sample.
*Significantly different from not treated (NT) cells. Significance was set at p<0.05.

## Slide 5
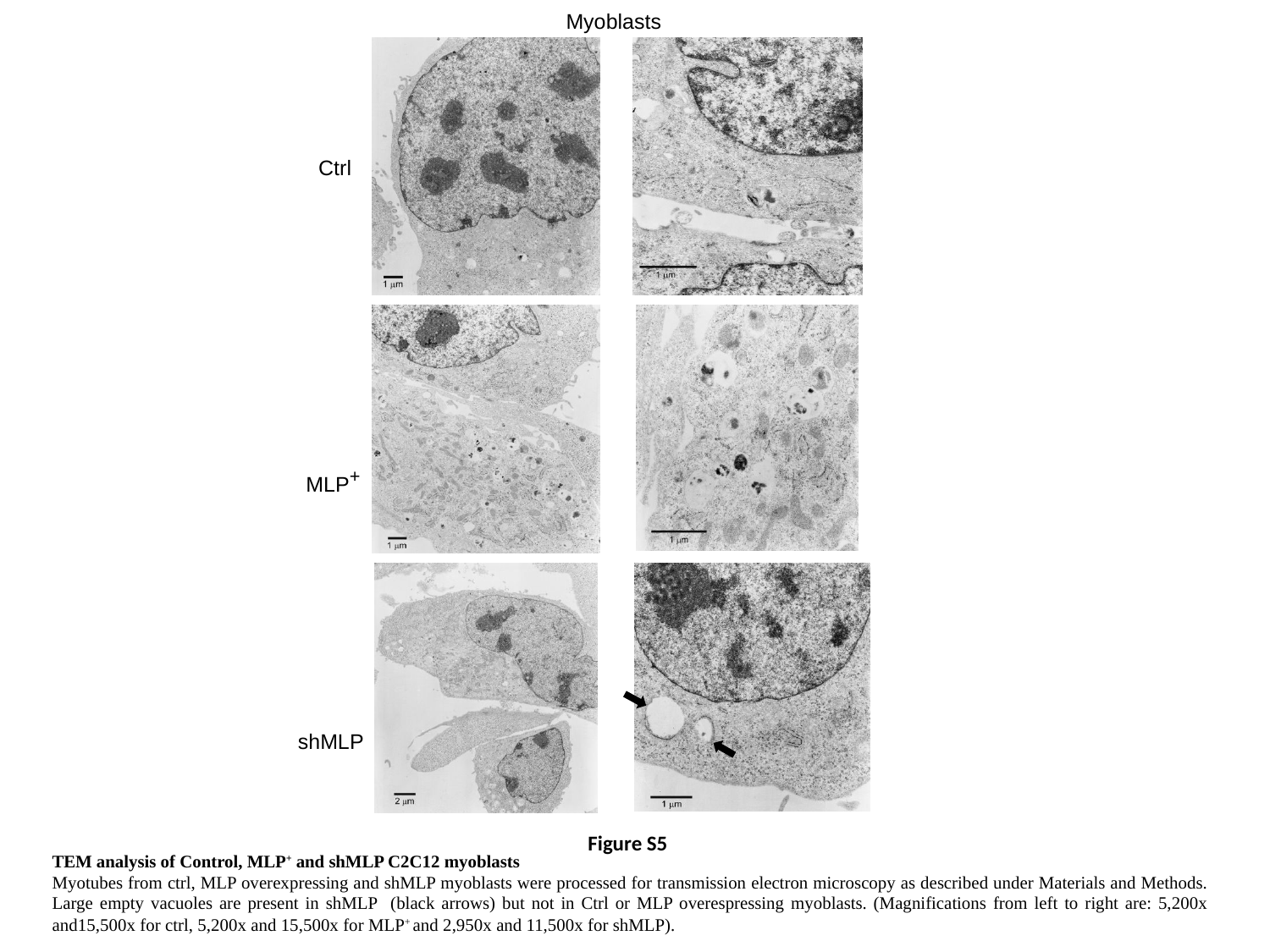

Myoblasts
Ctrl
MLP+
shMLP
Figure S5
TEM analysis of Control, MLP+ and shMLP C2C12 myoblasts
Myotubes from ctrl, MLP overexpressing and shMLP myoblasts were processed for transmission electron microscopy as described under Materials and Methods. Large empty vacuoles are present in shMLP (black arrows) but not in Ctrl or MLP overespressing myoblasts. (Magnifications from left to right are: 5,200x and15,500x for ctrl, 5,200x and 15,500x for MLP+ and 2,950x and 11,500x for shMLP).

## Slide 6
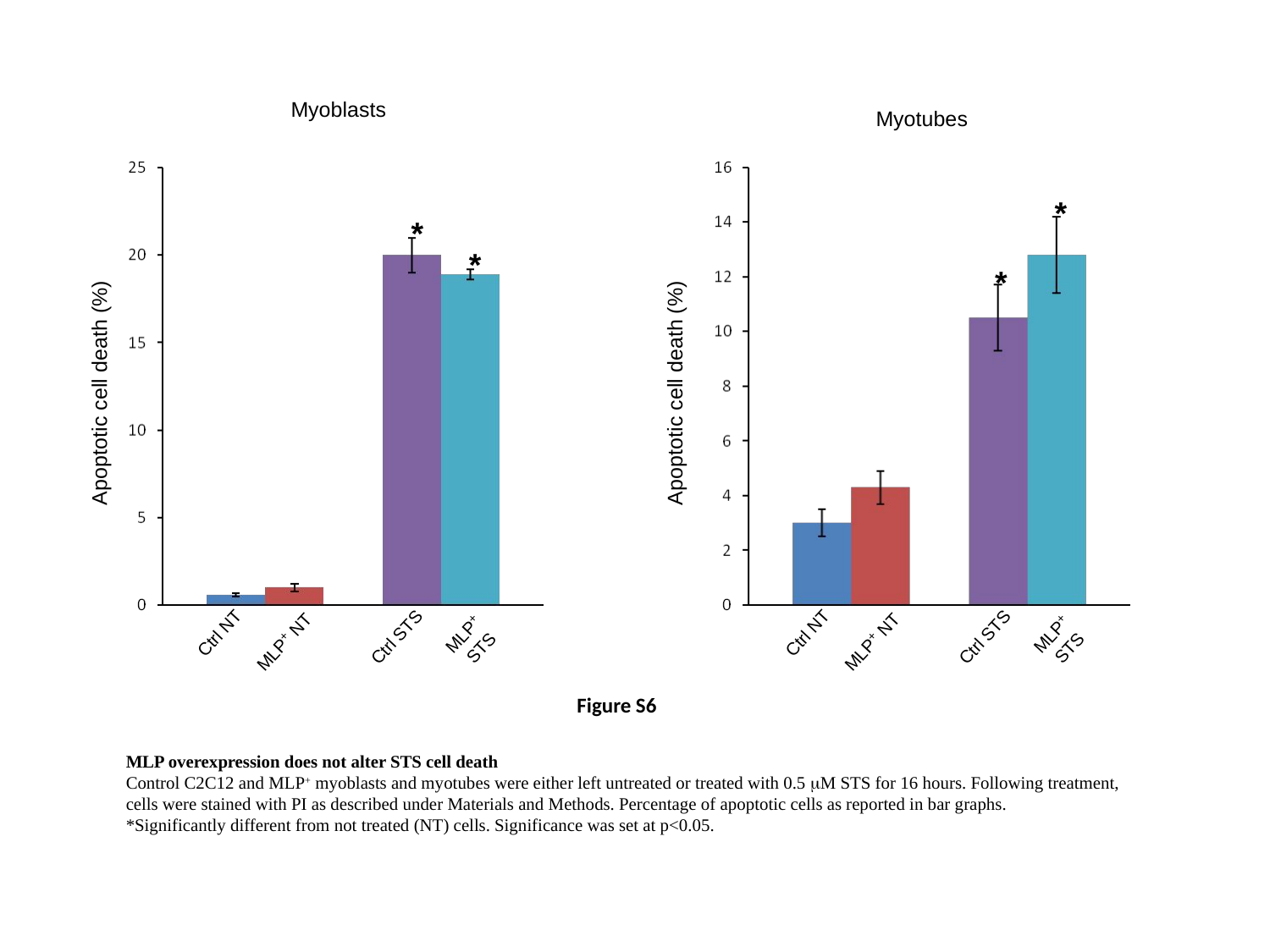

Myoblasts
Myotubes
*
*
*
*
Apoptotic cell death (%)
Apoptotic cell death (%)
MLP+
STS
MLP+
STS
Ctrl NT
Ctrl NT
Ctrl STS
Ctrl STS
MLP+ NT
MLP+ NT
Figure S6
MLP overexpression does not alter STS cell death
Control C2C12 and MLP+ myoblasts and myotubes were either left untreated or treated with 0.5 μM STS for 16 hours. Following treatment,
cells were stained with PI as described under Materials and Methods. Percentage of apoptotic cells as reported in bar graphs.
*Significantly different from not treated (NT) cells. Significance was set at p<0.05.
